# Initial Evaluation of Safety and Immunomodulatory Potential of Dietary Supplementation with Mangosteen Pericarp Extract for Sustainable Meat Production in Native Crossbred Chickens

**DOI:** 10.3390/life14111481

**Published:** 2024-11-14

**Authors:** Phruedrada Kaewtui, Chompunut Lumsangkul, Apinya Satsook, Korawan Sringarm, Chaiwat Arjin, Wanaporn Tapingkae, Pimporn Khamtavee, Orranee Srinual, Montri Punyatong, Kiattisak Huanhong, Peerawit Chongrattanameteekul, Natpasit Rattaworapanit, Thanawut Mangkang, Raktham Mektrirat

**Affiliations:** 1 Department of Animal and Aquatic Sciences, Faculty of Agriculture, Chiang Mai University, Chiang Mai 50200, Thailand; phruedrada_k@cmu.ac.th (P.K.); apinya.satsook@cmu.ac.th (A.S.); korawan.s@cmu.ac.th (K.S.); chaiwat.arjin@cmu.ac.th (C.A.); wanaporn.t@cmu.ac.th (W.T.); pimporn.k@cmu.ac.th (P.K.); orranee.s@cmu.ac.th (O.S.); montri.pun@cmu.ac.th (M.P.); kiattisak_huanhong@cmu.ac.th (K.H.); 2 Department of Animal Science, National Chung Hsing University, Taichung 40227, Taiwan; 3 Veterinary Academic Office, Faculty of Veterinary Medicine, Chiang Mai University, Muang, Chiang Mai 50100, Thailand; peerawit_ch@cmu.ac.th (P.C.); natpasit_r@cmu.ac.th (N.R.); r13629018@ntu.edu.tw (T.M.); 4 Department of Veterinary Medicine, School of Veterinary Medicine, National Taiwan University, Taipei 10617, Taiwan; 5 Chinese-Thai Cooperation Laboratory of Traditional Chinese Veterinary Medicine and Techniques, Faculty of Veterinary Medicine, Chiang Mai University, Chiang Mai 50100, Thailand; 6 Center of Excellence in Pharmaceutical Nanotechnology, Faculty of Pharmacy, Chiang Mai University, Chiang Mai 50200, Thailand

**Keywords:** feed additive, growth performance, meat quality, mangosteen, poultry, sustainability, food production, food security

## Abstract

The utilization of mangosteen biomass not only solves environmental problems but also raises the value of agricultural waste. The current study aimed to evaluate the potential of mangosteen pericarp extract (MPE) for enhancing the immunity and productivity of Thai native crossbred chickens on-farm. A total of 180 three-week-old chickens were divided into negative control and supplemented groups, with 1000 mg MPE/kg of diet. The safety of MPE was further confirmed by the absence of noticeable differences in mortality and biochemical parameters during the entire study period. The MPE-supplemented group displayed significant differences in the relative transcription levels of IL-10 compared to the basal diet group (*p* ≤ 0.01). Preslaughter body weight, average daily gain, and carcass weight in the MPE-supplemented group were higher than those in the basal diet group (*p* ≤ 0.05). Furthermore, MPE supplementation improved meat quality by enhancing the nutritional composition of protein and fat (*p* ≤ 0.05), as well as improving water-holding capacity, lowering boiling, and lowering grilling losses (*p* ≤ 0.01). These findings indicate that MPE can be an effective supplement for enhancing flock immunity, growth performance, and meat quality in poultry. This contributes to more sustainable agriculture and food security within agroecosystems.

## 1. Introduction

The rapid growth rate of the human population poses a concern over the sustainability of the ecosphere [1]. One major problem is the world’s limited resources, which have worsened equity among the population, leading to increased poverty and reduced access to quality food [2]. Therefore, ensuring a steady supply of protein is essential for maintaining food security on our planet. Poultry has the highest meat conversion rates compared to other terrestrial livestock. It also requires the least amount of water and land, resulting in a smaller environmental footprint [3,4,5]. Poultry also serves as a highly cost-effective source of meat protein. In addition, the consumption of poultry meat does not conflict with any major religious beliefs. Recently, many consumers have started to pay more attention to the food they eat, focusing on nutrients and seeking out foods that are beneficial to their health.

Interestingly, native chickens are typically healthier and exhibit better tolerance to local environmental conditions [6,7]. Beyond their nutritional benefits, native chickens offer local communities a chance to generate sustainable income. Consequently, the importance of native chickens has increased significantly in several Asian countries, including Thailand. Thai native chickens, which are primarily raised by small-scale farmers, exhibit non-uniform sizes, inconsistent meat quality, low final weight, and daily weight gain [8]. Consequently, a limited portion of this demand is being satisfied. Recently, efforts to improve production have focused on morphological selection, physical properties, and crossbreeding [9]. The Pradu Hang Dam × Hubbard JA57Ki crossbred native chicken was developed to improve production and meat quality. However, adding additional nutrition is a further great approach to upgrading from support to economically sustainable semi-commercial productivity. It is crucial for successful cultivation, and adding phytogenic feed additives is expected to enhance the growth, health, and overall well-being of chickens [10,11].

Mangosteen (*Garcinia mangostana* L.) is a tropical fruit native to Southeast Asia that has gained global popularity for its unique flavor and health benefits. Thailand is the largest producer and exporter of mangosteen in Southeast Asia, accounting for around 85%, followed by Indonesia, Malaysia, and the Philippines [12]. Although the pericarp of mangosteen is usually discarded, leading to significant agricultural waste and raising environmental concerns, many studies have suggested that the mangosteen pericarp is actually rich in bioactive phytochemicals [13,14]. Studies on the biological activity of mangosteen pericarp have found that it possesses antibacterial, anticoccidial, antioxidant, and anti-inflammatory properties [15,16]. The study of mangosteen as a feed additive was performed in various study species, including swamp buffalo [17], goat [18], fishes [19], and broilers [20]. Unfortunately, the reports on Thai native crossbred chicken is very limited.

Therefore, this preliminary study aimed to evaluate the efficacy of dietary supplementation with the mangosteen pericarp extract on the productive performance, meat quality, and immune response of Thai native crossbred chickens. We hypothesize that the inclusion of mangosteen pericarp in their diet will not only enhance growth performance and meat quality but also improve immune-related gene expression, contributing to better overall flock health.

## 2. Materials and Methods

### 2.1. Animals and Husbandry

A total of 180 (3-week-old) Thai native crossbred chickens (Pradu Hang Dam) were obtained from Agriculture Innovation Research, Integration, Demonstration and Training Center, Chiang Mai University, Chiang Mai, Thailand. The chickens were raised in an open house with a pen measuring 2 × 2 m, accommodating 15 birds. The temperature and humidity were 29–30 °C and 55–60%, respectively. The floor was covered with rice husks as litter. Light was available all night. Throughout the 9 weeks of the experiment, the chickens had free access to food and drinking water ad libitum. The experiment followed the protocols authorized by the Animal Ethics Committee of the Faculty of Agriculture, Chiang Mai University (Ethic Permit No. RAGIACUC012/2565).

### 2.2. Plant Extract Composition and Total Phenolic Biomarker Analysis

Powdered extract of mangosteen pericarp (MPE) was obtained from Specialty Natural Products Co., Ltd., Chonburi, Thailand. The powdered MPE was analyzed for chemical composition following the AOAC recommendation, total phenolic content (TPC), using the Folin–Ciocalteu method [21]. The extract was combined with Folin–Ciocalteu reagent and a 7.5% (*w*/*v*) sodium carbonate (Na_2_CO_3_) solution. After a 60 min incubation period, a calibration standard for gallic acid was established using a UV–Vis spectrophotometer (SPECTROstar Nano, BMG LABTECH, Ortenberg, Germany). The TPC of the extract was quantified in milligrams of gallic acid per gram.

### 2.3. Dietary Preparation and Feeding

A basal diet consisted of a commercial standard broiler diet, and the compositions are detailed in Table 1. The MPE powder was evenly sprinkled over a small portion of the basal feed and thoroughly mixed. Subsequently, this mixture was carefully integrated into the required amount of feed, ensuring complete homogenization using a feed mixer machine (Model MI1HP3V01, Siam Farm Service Co., Ltd., Lampang, Thailand) to achieve the final concentrations of MPE at 1000 mg/kg of diet. The chickens were randomly assigned to 2 groups, with 6 replicates per group and 15 chickens per replicate. The control group received a standard basal diet, while the supplement group received diets with MPE.

### 2.4. Safety Evaluations

Mortality, behavioral expression, physical appearance, and any signs of illness were monitored daily throughout the study period. The whole blood sample was randomly collected from one bird per replicate (n = 12). Blood biochemical parameters, including alanine aminotransferase (ALT), alkaline phosphatase (ALP), aspartate aminotransferase (AST), total bilirubin (TBIL), direct bilirubin (DBIL), indirect bilirubin (IBIL), total protein (TP), albumin (ALB), and globulin (GLB), were measured using an automated BX-3010 analyzer (Sysmex, Kobe, Japan).

### 2.5. Analysis of Immune-Related Gene Expressions

At the end of the experiment, hepatic tissues were randomly collected from one bird per replicate (n = 12) and immediately stored at −20 °C until further examination. The RNA extraction was carried out according to the manufacturer’s instructions (Invitrogen, PureLink™ RNA Mini Kit, Waltham, MA, USA). The purity and yield of the RNA were measured. Reverse transcription was performed using the Bio-Rad iScript™ RT Supermix cDNA synthesis kit (Bio-Rad, Hercules, CA, USA). qPCR reaction was performed using CFX Connect™ Real-Time PCR System (Bio-Rad, Hercules, CA, USA) and iTaq Universal SYBR Green supermix 2× (Bio-Rad, USA) with specific gene primers including IL-1β, IL-10, and TNF-α genes [22]. The immune-related genes expression levels were quantified relative to the housekeeping gene by the 2−ΔΔCt method and standard curve.

### 2.6. Measurement of Flock Productivity

Chickens were randomly weighed from two birds per replicate (n = 24) at both the initial phase (week 3) and the final phase (week 12). The feed intake and chicken mortality were recorded daily throughout the study period. The average daily feed intake (ADFI), average daily gain (ADG), and feed conversion ratio (FCR) were calculated. In addition, the European Broiler Index (EBI) and Production Efficiency Factor (PEF) were also generated to provide further information regarding the growth performance of the chickens [23].

### 2.7. Characterization of Carcasses

At the twelfth week, two chickens were randomly selected from each replicate (n = 24) and slaughtered by cervical disarticulation. The eviscerated carcass and dressed weights were recorded after removing all the feathers and visceral organs. The edible viscera, including the liver, gizzard, heart, and spleen, were collected and weighed immediately. The breasts, thighs, drumsticks, wings, neck, and legs were all separated and weighed. The carcass and relative organ weights were also calculated based on live weight percentage.

### 2.8. Measurement of Meat Quality

After slaughter, chicken meat from one bird per replicate (n = 12) was collected. The breast and thigh muscles were measured for initial pH value (45 min post-slaughter), final pH (24 h post-slaughter), color, water-holding capacity (WHC), shear force, and chemical composition. The pH values were measured using a pH meter (Shenzhen Jige Electromechanical Equipment Co., Ltd., Shenzhen, China). The CR-10 Plus chromameter (Zibo Diye Instrument Equipment Co., Ltd., Zibo, China) was used to measure the color lightness (L*), yellowness (b*), and redness (a*) of pectoral and leg muscle samples. Muscular protein and fat content were analyzed following the AOAC recommendation [24,25]. The protein content in the meat was quantified using the Kjeldahl method with the Kjeltec System 8400 (FOSS NIRSystems Inc., Hillerød, Denmark). The fat content in the meat was assessed utilizing the Soxhlet extraction apparatus (Foss Soxtec Avanti 2055; Foss, Hoganas, Sweden). After carcass evaluation, the muscular breast and thigh samples were stored for 24 h in a refrigerator before being tested for water-holding capacity of drip loss and cooking loss as well as shear force, according to standard methods [26]. To determine drip loss (%), meat samples were placed in sealed plastic bags and refrigerated at 4 °C for 24 h. The disparities between the initial and final weights of the samples were computed and shown as relative weights to the initial weight. The measurement of cooking loss (%) was performed following the methodology established by [26]. Fresh muscle samples, measuring 4 × 3 × 1 cm^3^, were weighed, placed in sealed plastic bags, and cooked until an internal temperature of 70 °C was reached, which required approximately 15 min in a water bath maintained at 80 °C. Thereafter, the slices were chilled in water, desiccated, and reweighed. The cooking loss percentage was calculated using the formula: (starting weight − final weight)/initial weight × 100%.

### 2.9. Statical Analysis

The data were analyzed using descriptive statistics, which included proportions and means with standard deviations. The distribution of continuous variables was assessed through Q-Q plots. The association between continuous variables of breasts and thighs in the MPE-supplemented group was assessed using Pearson’s correlation coefficients. Further analysis was conducted using the unpaired *t*-test to compare the means between groups. Differences were considered statistically significant when the probability of the two-tailed test was less than 5% (*p* ≤ 0.05). Statistical analysis was performed using the R statistics package (RStudio, Boston, MA, USA) and GraphPad Prism software, version 9.0 (San Diego, CA, USA).

## 3. Results

### 3.1. Proximate Composition and Total Phenolic Biomarker of MPE

The Integrated Taxonomic Information System (ITIS) report provides the taxonomic serial number 21484. Fresh mangosteen fruit was collected from Chonburi, Thailand, at the coordinates 13°21′40.1148″ N and 100°59′4.8228″ E. The proximate composition of the MPE powder, as detailed in Table 2, reveals that it comprises 4.54% crude fat, 0.51% moisture, 0.44% protein, and 0.22% ash. The total phenolic content of MPE was 3.60 mg of gallic acid equivalents per gram (mg GAE/g).

### 3.2. Safety

The lethality assessment of chickens supplemented with a 1000 mg MPE/kg diet demonstrated that no deaths occurred in any animal over the 12-week period. No noticeable changes in general appearance or behavior throughout the research period were also observed. For blood biochemical analysis (Table 3), no significant differences were observed between the basal diet group and the MPE-supplemented group for all serum protein and lipid parameters (*p* > 0.05). Moreover, supplementation with MPE did not affect serum enzymes, including aspartate transaminase, alanine transaminase, and alkaline phosphatase (*p* > 0.05)

### 3.3. Flock Immunity

**Figure 1:**
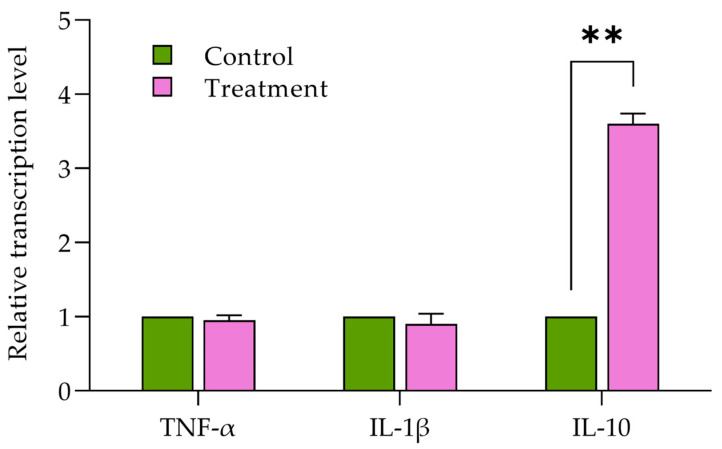
Bar graph illustrating relative transcript levels of immune-related genes in liver tissue in Thai native crossbred chickens fed a basal diet with and without supplementation of a 1000 mg MPE/kg diet. This figure compares the expression levels of tumor necrosis factor-α, interleukin-1β, and interleukin-10, comparing the negative control and the MPE-supplemented groups. Error bars represent the standard deviations of the means for each group. Data were analyzed using the Student’s *t*-test, with an asterisk (**) indicating a statistically significant difference between groups (*p* ≤ 0.01).

The effects of MPE on the relative expression of genes involved in the immunity in the liver of chickens are presented in Figure 1. The results demonstrate that the relative immune-related gene expressions of proinflammatory cytokines, including IL-1β and TNF-α genes, in the group supplemented with 1000 mg MPE/kg diet have no significant differences compared to the basal diet group (*p* = 0.42). Whereas the MPE-supplemented group displays significant differences in the relative transcription levels of the anti-inflammatory cytokine of IL-10 compared to the basal diet group (*p* ≤ 0.01).

### 3.4. Growth Performance and Economic Viability

**Figure 2:**
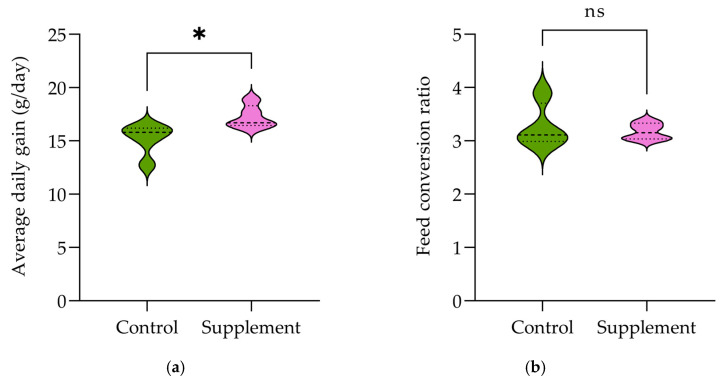
Violin graphs illustrating growth performance in Thai native crossbred chickens fed a basal diet with and without supplementation of 1000 mg MPE/kg diet. The graphs depict the distribution of (**a**) average daily gain and (**b**) feed conversion ratio. Statistical analysis was conducted using the Student’s *t*-test, with an asterisk (*) indicating a statistically significant difference between groups (*p* ≤ 0.05). (ns) indicating no statistically significant difference between groups (*p* > 0.05).

No significant difference was found between the control group and the supplement group in initial weight (*p* > 0.05). The effects of MPE on growth performance and carcass traits in Thai native crossbred chickens are presented in Table 4. Preslaughter body weight in the MPE-supplemented diet was significantly improved (*p* = 0.04). Additionally, the growth performance indicator of average daily gain in the MPE-supplemented group (17.24 ± 1.06 g) was higher than that of the basal diet group (15.21 ± 1.44 g) (Figure 2a), whereas there was no significant effect of dietary supplementation with and without MPE on the feed conversion ratio (control, 3.27 ± 0.42; supplement, 3.17 ± 0.16) (Figure 2b), the European broiler index, and the production efficiency factor of chickens between the basal diet group and the MPE-supplemented group (*p* > 0.05).

### 3.5. Carcass Traits

**Figure 3:**
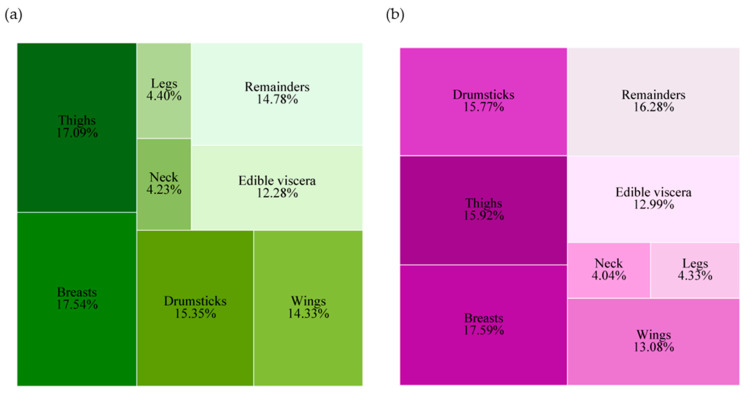
Treemap chart illustrating the proportion of carcass traits and related visceral organs in Thai native crossbred chickens. (**a**) Data from the basal diet group and (**b**) data from the group supplemented with 1000 mg MPE/kg diet, in which the variables are grouped into eight classes, each represented by different colors, including six carcass parts, one edible visceral organ, and one reminder.

For carcass traits, the chickens fed the diet with MPE exhibited a greater carcass weight (*p* = 0.04); however, the percentage of dressing weight in the MPE-supplemented group did not differ statistically compared to the control group (*p* > 0.05) (Table 4). Overall, the data given in Figure 3 demonstrate that dietary MPE supplementation had no effect on the carcass characteristics investigated, which included breast muscle, thigh muscle, drumsticks, wings, neck, and legs (*p* > 0.05). MPE supplementation also had no effect on any of the characteristics of edible viscera, including the liver, gizzard, heart, and spleen (*p* > 0.05).

### 3.6. Muscular Chemical Composition and pH Values

The basic chemical composition of chicken breast and thigh meats is presented in Table 5. The results show a lower fat content in the breasts (0.75 ± 0.02) and thighs (3.82 ± 0.01) of the MPE-supplemented group compared to the control group, which had a fat content of 1.15 ± 0.01 in the breast and 4.53 ± 0.01 in the thighs (*p* ≤ 0.01). Moreover, the protein content in the breast meat of the MPE-supplemented group (24.29 ± 0.04) was higher than that of the control group (23.08 ± 0.26) (*p* = 0.02). The water content in the breast and thigh meats of both the MPE-supplemented group and the control group was not statistically different (*p* > 0.05).

The average initial pH and ultimate pH in the breast and thigh meats of the MPE-supplemented group seem higher than those of the control group (Table 6). Additionally, no significant difference was observed in the pH difference between the ultimate pH and the initial pH in both types of meat (*p* > 0.05). Moreover, the MPE-supplemented group exhibited a very strong positive correlation for the initial pH value (*r* = 0.87) and a weak positive correlation for the ultimate pH value (*r* = 0.19) in breasts and thighs (*p* > 0.05) (Table 6).

### 3.7. Muscular Color

**Figure 4:**
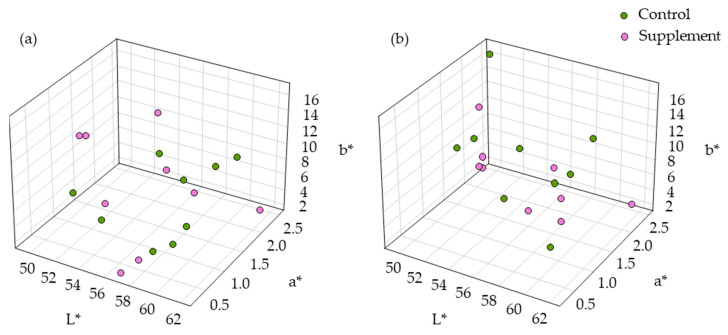
Three-dimensional scatter plot of muscular color in Thai native crossbred chickens. The scatter plot illustrates the relationship between lightness, redness, and yellowness values for (**a**) breast and (**b**) thigh meat samples from chickens fed a basal diet with and without supplementation of 1000 mg MPE/kg diet. The L* axis represents lightness, the a* axis represents redness, and the b* axis represents yellowness.

Both the breast and thigh meats showed no significant differences in muscular colors, specifically lightness (L*), redness (a*), and yellowness (b*) values, between the control and the MPE-supplemented groups (*p* > 0.05). The scatter plot of visual appearance using L*, a*, and b* coordinates for the control and MPE-supplemented groups is presented in Figure 4. Moreover, a positive correlation was observed for yellowness (*r* = 0.60) and redness (*r* = 0.10) in both breasts and thighs, while a medium negative correlation (*r* = −0.40) was noted for lightness within the MPE-supplemented group (*p* > 0.05) (Table 6).

### 3.8. Water-Holding Capacity and Shear Force

**Figure 5:**
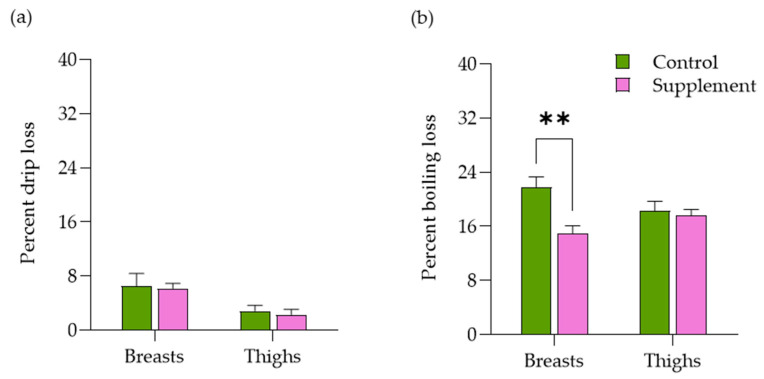
Bar graphs illustrating the water-holding capacity of chicken meats in Thai native crossbred chickens fed a basal diet with and without supplementation of 1000 mg MPE/kg diet. The water-holding capacity for (**a**) drip loss and (**b**) boiling loss in breast and thigh muscles is presented. Statistical analysis was conducted using the Student’s *t*-test, with an asterisk (**) indicating a statistically significant difference between groups (*p* ≤ 0.01).

For muscular breasts, boiling loss (14.96 ± 1.12%) in the MPE-supplemented group was lower than that of the control group (21.81 ± 1.32%) (*p* ≤ 0.01) (Figure 5). However, both breast and thigh meats showed no significant differences in muscular drip loss between the control and MPE-supplemented groups (*p* > 0.05). For the MPE-supplemented group, drip loss (*r* = 0.10) showed a weak positive correlation between breasts and thighs (*p* > 0.05), while boiling loss (*r* = −0.46) indicated a medium negative correlation between these two muscle groups *(p* > 0.05) (Table 6). Both breasts’ (27.69 ± 35.49) and thighs’ (27.54 ± 0.64) shear forces in the MPE-supplemented group did not statistically differ from the control group (breasts, 28.86 ± 3.40; thighs, 25.47 ± 1.74) (*p* > 0.05). In addition, the shear forces observed in the MPE-supplemented group (r = 0.91) suggested a very strong positive correlation between the breasts and thighs (*p* > 0.05).

## 4. Discussion

The pericarp of the mangosteen accounts for around sixty percent of the fruit and has become agricultural waste [13]. Utilizing the disposal of mangosteen pericarp not only addresses ecological problems but also increases the value of this agricultural by-product. However, the biomarkers of total phenolic content from dried MPE powder should be measured to ensure the quality of MPE as a feed additive in chicken diets. In this study, the gallic acid equivalents per gram (GAE/g) for the total phenolic content of the MPE from Thailand agreed with prior studies, which found values of 6.7 mg GAE/g for the n-butanol fraction and 4.1 mg GAE/g for the water fraction [27]. Moreover, the polyphenolic xanthonoid content of α-mangostin in methanol (403.9 ± 13.1 mg/g of extract) and aqueous extracts (2.5 ± 0.4 mg/g of extract) of mangosteen pericarp was previously reported [28]. The phytochemical composition of MPE is determined by the plant ontogenesis and different techniques for extraction and solvents [29].

The safety of MPE was confirmed by the absence of mortality or evidence of intoxication after daily repeated exposure to 1000 mg of MPE/kg dietary regimens in Thai native crossbred chickens for the 12-week feeding trial. This finding is consistent with previous studies indicating that acute and subchronic toxicity tests showed no effects on behavior and health condition in Wistar rats with a median lethal dose (LD_50_) ≥ 2000 mg/kg body weight and No Observed Adverse Effect Level (NOAEL) 1000 mg/kg [30,31]. Additionally, the chronic toxicity test of MPE in Wistar rats (10–1000 mg/kg/day for 6 months) demonstrated that MPE did not produce any pharmacotoxicity signs [32]. To evaluate the safety profile of α-mangostin, several general toxicity studies have also been previously reported [33]. Furthermore, the finding of normal blood chemistry supported the safety of MPE, as the absence of significant differences in hepatic enzymes and serum proteins between the control and MPE-supplemented groups provides important insight into the lack of adverse effects on hepatic function homeostasis [34,35].

In this study, the relative expression of the IL-10 in the liver of chickens fed a diet containing 1000 mg of MPE/kg was higher than that in chickens fed a basal diet. IL-10 is an extremely effective anti-inflammatory cytokine that plays a crucial role in the regulation from the autoimmunity and the inflammatory responses during infection with viruses, bacteria, fungi and parasites [36]. The anti-inflammatory effect of mangosteen pericarp is associated with xanthones, which modulate pro- and anti-inflammatory cytokines and mediators. Additionally, the xanthone derivatives exhibited marked anti-inflammatory actions, resulting in a significant decrease in serum TNF-α and an increase in serum IL-10 in both computational and biological studies [37]. A prior study indicated that the heterophil-to-lymphocyte ratios (H/L ratio) tended to be decreased in quails that consumed MPE in drinking water during heat stress [38]. Another previous study demonstrated that the mangosteen rind powder at 66 and 100 g/kg of broiler feed enhances the immune system by increasing the peripheral blood counts and lymphocyte concentration in the cloacal bursa, as well as the formation of lymphatic organs [39].

For livestock production, there is potential for using phytogenic bioactive substances to mitigate oxidative stress and inflammation in poultry [40,41]. The inflammation and prolonged stress might trigger lipid peroxidation, protein oxidation, destruction of DNA, and, eventually, cellular death. These effects contribute to negatively impact the animal performance, health, and welfare [42]. In addition, the preslaughter body weight and the average daily gain in the MPE-supplemented group in this study were significantly improved. This result aligns with previous studies indicating that broiler performance improves with dietary supplementation of mangosteen peel or a combination of mangosteen peel and ginger rhizome [43]. Moreover, Cihateup duck performance can be enhanced by supplementing the diet with a combination of mangosteen peel flour and turmeric flour [44]. Interestingly, plant extracts rich in diverse polyphenolic compounds have garnered significant attention as alternative antibiotic growth promoters in broiler production [45,46]. Previous studies have demonstrated that xanthones enhance the structure of intestinal villi in broiler chickens, facilitating nutrient absorption and inhibiting the growth of pathogenic bacteria in the intestines [47]. Furthermore, Garcinone D confers protection against intestinal epithelial barrier dysfunction through a dual mechanism involving the activation of both the aryl hydrocarbon receptor (AhR) and nuclear factor erythroid 2-related factor 2 (Nrf2) signaling pathways, as previously reported [48]. Therefore, this improvement in nutrient absorption leads to better growth and optimal carcass weight.

In the present investigation, the carcass weight in the MPE-supplemented group was significantly larger than that of the control group. Both the breast and thigh meats showed no significant differences in the ultimate pH, the muscle color, and the drip loss between the control and MPE-supplemented groups. Furthermore, the association between final pH, lightness, and drip loss in boiler meat has been previously documented [49]. However, the boiling loss of the breasts in the MPE-supplemented group was lower than in the control group. This finding partially aligns with a previous study indicating that the enhancement of meat quality occurred by reducing total water loss and drip loss in the muscles of broilers fed MPE [50]. Regarding meat quality, the muscular nutritional composition yield in the MPE-supplemented group showed a lower fat content in both the breasts and thighs, along with a higher protein content in the breasts compared to the control group. This finding is consistent with a previous study on Sentul chickens, indicating that the cholesterol content in the meat decreased in proportion to the amount of xanthones [51]. Additionally, α-Mangostin not only enhances the activity of the lipoprotein lipase enzyme, which facilitates the breakdown of Very Low-Density Lipoprotein (VLDL), but also promotes the secretion of pancreatic lipase and α-amylase in the anti-obesity mechanism [52]. The muscular chemical composition is an essential nutritional parameter for chicken meat and also currently has considerable effects on meat consumption [53]. In addition, the perceived quality of native chicken meat has been related to muscle fiber properties, containing higher protein contents, but rather lower fat and ash contents compared with broiler muscles [54,55].

For optimal gains of high profits from meat chicken farming, it is critical to achieve productive and economic efficiency [56]. Interestingly, the average daily gain and the carcass weight in the MPE-supplemented group were significantly greater than those of the control group in this study. There was no significant effect of the dietary supplementation of MPE on the European broiler index and the production efficiency factor; however, both economic parameters in the MPE-supplemented group appeared to be higher than those of the control group. These findings agree with previous investigations that reported that the supplementation of medicinal plants contributed to a higher economic efficiency index as well as lower costs in broiler production [57]. Furthermore, phytogenic feed additives could be employed in chicken production as an effective alternative approach to benefiting animals, the environment, and customers.

## 5. Conclusions

The present research highlights that the supplementation of 1000 mg of mangosteen pericarp extract per kg of diet in Thai native crossbred chickens had positive effects on flock immunity by increasing the levels of IL-10. Moreover, flock production exhibited a satisfactory average daily gain, preslaughter body weight, and carcass weight. The MPE supplementation also resulted in improvements of the meat quality, including the nutritional composition of protein and fat, as well as the water-holding capacity. These results suggest that the use of MPE presents a promising approach to achieving more sustainable and efficient poultry production in Thai native crossbred chickens. However, further research may be necessary to fully understand the various amounts of MPE required for optimizing the dosage of this phytogenic feed additive.

## Figures and Tables

**Table 1:** Basal diet ingredients and chemical composition on a dry matter basis.

Items	4–6 Weeks	7–12 Weeks
Ingredient (g/kg feed)		
Corn meal	70.0	57.5
Rice bran	0	7.5
Full fat soybean meal	0	2.5
Soybean meal 44%	20.5	19.25
Meat meal 50%	2.5	2.5
Limestone	1.0	2.5
Calcium carbonate	0	4.74
Monopotassium phosphate 22%	1.05	1.75
Premix ^1^	0.25	0.25
Methionine	0.09	0.15
Toxin binder	0.1	0.05
Salt	0	0.2
Multi protein plus 68%	4.5	1.1
Phytase	0.01	0.01
Analyzed composition (% dry matter basis)		
Moisture	12.23	9.78
Ash	6.79	11.91
Crude protein	22.22	18.02
Crude fiber	4.56	3.82
Crude fat	5.15	4.59
Metabolizable Energy (Kcal/Kg)	2964.92	3081.65

^1^ Premix: The supplied vitamin–mineral premix contains the following per kilogram of diet: 15,000 IU vitamin A, 3000 IU vitamin D3, 25 IU vitamin E, 5 mg vitamin K3, 2 mg vitamin B1, 7 mg vitamin B2, 4 mg vitamin B6, 25 mg vitamin B12, 11.4 mg pantothenic acid, 35 mg nicotinic acid, 1 mg folic acid, 15 μg biotin, 250 mg choline chloride, 1.6 mg Cu, 60 mg Mn, 45 mg Zn, 80 mg Fe, 0.4 mg I, and 0.15 mg Se.

**Table 2:** Percentage total of proximate composition of mangosteen pericarp extract.

Parameters	Composition (g/100 g Dryweight Basis)
Dry matter (%)	96.50
Moisture (%)	0.51
Ash (%)	0.22
Crude protein (%)	0.44
Crude fat (%)	4.54

**Table 3:** Blood biochemical profiles in Thai native crossbred chickens fed a basal diet with and without supplementation of 1000 mg MPE/kg diet.

Parameters	Experimental Group		*t*-Statistic	*p*-Value
	Control (n = 12)	Supplement (n = 12)		
Aspartate transaminase (U/L)	264.33 ± 56.04	252.33 ± 17.59	0.29	0.79
Alanine transaminase (U/L)	0.97 ± 0.05	0.93 ± 0.05	0.71	0.52
Alkaline phosphatase (U/L)	1721.33 ± 383.75	1621 ± 354.55	0.27	0.80
Total protein (mg/dL)	3.87 ± 0.39	3.80 ± 0.14	0.23	0.83
Albumin (mg/dL)	2.53 ± 0.34	2.43 ± 0.12	0.39	0.72
Globulin (mg/dL)	1.33 ± 0.17	1.37 ± 0.09	0.24	0.82
Total bilirubin (mg/dL)	0.18 ± 0.01	0.20 ± 0.01	1.25	0.28
Direct bilirubin (mg/dL)	0.02 ± 0.01	0.02 ± 0.01	0.35	0.74
Indirect bilirubin (mg/dL)	0.17 ± 0.02	0.18 ± 0.02	0.69	0.53
Total cholesterol (mg/dL)	91.00 ± 17.96	92.00 ± 15.77	0.06	0.96
Triglyceride (mg/dL)	53.00 ± 17.8	101.67 ± 37.95	1.64	0.18

**Table 4:** Growth performance, carcass weight, and economic viability in Thai native crossbred chickens fed basal diet with and without supplementation of 1000 mg MPE/kg diet.

Parameters	Experimental Group		*t*-Statistic	*p*-Value
	Control (n = 24)	Supplement (n = 24)		
Initial weight (g)	158.79 ± 3.46	157.11 ± 3.36	0.50	0.63
Final weight (g)	1116.82 ± 95.38	1243.00 ± 66.94	2.42	0.04
Average daily feed intake (g)	49.17 ± 1.46	54.63 ±1.58	5.07	<0.01
Carcass weight (g)	766.11 ± 55.48	881.63 ± 66.57	4.36	<0.01
Dressing weight (%)	61.90 ± 1.59	62.53 ± 1.61	0.93	0.37
European broiler index	47.58 ± 9.98	53.16 ± 4.08	1.04	0.34
Production efficiency factor	0.67 ± 0.12	0.63 ± 0.05	1.04	0.34

**Table 5:** Chemical composition and pH values of muscular breasts and thighs from Thai native crossbred chickens fed basal diet with and without supplementation of 1000 mg MPE/kg diet.

Muscle	Parameters	Experimental Group		*t*-Statistic	*p*-Value
		Control (n = 12)	Supplement (n = 12)		
Breast	Composition (%)				
	Water	1.71 ± 0.04	1.72 ± 0.01	0.24	0.83
	Protein	23.08 ± 0.26	24.29 ± 0.04	6.56	0.02
	Fat	1.15 ± 0.01	0.75 ± 0.02	150.80	<0.01
	pH value				
	Initial pH	5.10 ± 0.22	5.76 ± 0.21	2.87	<0.01
	Ultimate pH	5.47 ± 0.09	5.50 ± 0.05	1.05	0.31
Thigh	Composition (%)				
	Water	1.07 ± 0.01	1.08 ± 0.02	2.20	0.16
	Protein	19.63 ± 0.12	19.21 ± 0.18	5.56	0.03
	Fat	4.53 ± 0.01	3.82 ± 0.01	50.66	<0.01
	pH value				
	Initial pH	6.28 ± 0.16	6.34 ± 0.10	1.29	0.21
	Ultimate pH	5.80 ± 0.08	5.80 ± 0.11	0.05	0.96

**Table 6:** Correlation coefficients of meat quality between muscular breasts and thighs from chickens fed with supplementation of 1000 mg MPE/kg diet.

Parameters	Muscle		Correlation Coefficient Value	*p*-value
	Breasts (n = 12)	Thighs (n = 12)		
Initial pH	5.77 ± 0.18	6.35 ± 0.11	0.87	0.13
Ultimate pH	5.51 ± 0.05	5.87 ± 0.08	0.19	0.81
Lightness	52.85 ± 1.04	61.56 ± 5.62	−0.40	0.60
Redness	1.04 ± 0.86	3.1 ± 1.87	0.10	0.90
Yellowness	6.06 ± 1.02	2.6 ± 1.63	0.60	0.40
Drip loss (%)	6.18 ± 0.72	2.28 ± 0.82	0.10	0.80
Boiling loss (%)	14.96 ± 1.12	17.59 ± 0.9	−0.46	0.54
Shear force (N)	47.46 ± 35.49	27.5 ± 0.64	0.91	0.27

## Data Availability

The data presented in this study are available on request from the corresponding author.
